# Network Analysis Reveals Putative Genes Affecting Meat Quality in Angus Cattle

**DOI:** 10.3389/fgene.2017.00171

**Published:** 2017-11-06

**Authors:** Raluca G. Mateescu, Dorian J. Garrick, James M. Reecy

**Affiliations:** ^1^Department of Animal Sciences, University of Florida, Gainesville, FL, United States; ^2^Department of Animal Science, Iowa State University, Ames, IA, United States

**Keywords:** Angus, GWAS, gene networks, meat quality, tenderness

## Abstract

Improvements in eating satisfaction will benefit consumers and should increase beef demand which is of interest to the beef industry. Tenderness, juiciness, and flavor are major determinants of the palatability of beef and are often used to reflect eating satisfaction. Carcass qualities are used as indicator traits for meat quality, with higher quality grade carcasses expected to relate to more tender and palatable meat. However, meat quality is a complex concept determined by many component traits making interpretation of genome-wide association studies (GWAS) on any one component challenging to interpret. Recent approaches combining traditional GWAS with gene network interactions theory could be more efficient in dissecting the genetic architecture of complex traits. Phenotypic measures of 23 traits reflecting carcass characteristics, components of meat quality, along with mineral and peptide concentrations were used along with Illumina 54k bovine SNP genotypes to derive an annotated gene network associated with meat quality in 2,110 Angus beef cattle. The efficient mixed model association (EMMAX) approach in combination with a genomic relationship matrix was used to directly estimate the associations between 54k SNP genotypes and each of the 23 component traits. Genomic correlated regions were identified by partial correlations which were further used along with an information theory algorithm to derive gene network clusters. Correlated SNP across 23 component traits were subjected to network scoring and visualization software to identify significant SNP. Significant pathways implicated in the meat quality complex through GO term enrichment analysis included angiogenesis, inflammation, transmembrane transporter activity, and receptor activity. These results suggest that network analysis using partial correlations and annotation of significant SNP can reveal the genetic architecture of complex traits and provide novel information regarding biological mechanisms and genes that lead to complex phenotypes, like meat quality, and the nutritional and healthfulness value of beef. Improvements in genome annotation and knowledge of gene function will contribute to more comprehensive analyses that will advance our ability to dissect the complex architecture of complex traits.

## Introduction

Increasing consumer demand for beef is an important strategic objective for the beef industry and recent studies suggest consumers have a strong focus on beef quality (Igo et al., 2013; Schroeder et al., 2013). Beef quality is largely communicated in terms of USDA quality grade as higher quality grade beef will contain more intramuscular fat, which improves flavor, juiciness, and positively influences tenderness (Koohmaraie et al., 2002). These properties have a large impact on the eating experience of the consumer, and consumer's eating satisfaction is the main driver of beef demand (Schroeder et al., 2013). However, in addition to eating satisfaction, other attributes including nutritional value, and healthfulness (fatty acid composition and mineral content) are important components of quality in the eyes of modern consumers.

All the components defining eating quality can be regarded as quantitative traits, controlled by many genes and impacted by environmental factors. Most component traits are difficult and expensive to measure and not available to measure until late in life or after the animal has been harvested. Such traits are difficult to improve through traditional phenotypic selection, but are ideal candidates for genomic selection if genetic markers explaining a large enough proportion of the variation can be identified. Warner-Bratzler Shear Force (WBSF, an objective measure of tenderness) and the intramuscular fat content (IMFC) were identified from an extensive set of carcass and meat composition traits to be the best predictors of eating quality (Mateescu et al., 2016). Those indicator traits are difficult to measure on live animals and DNA tests that can accurately identify cattle with superior genetics for WBSF and IMFC would be helpful. Knowledge of the genetics controlling these traits along with a precise understanding of the biological networks and interactions underlying the meat quality complex will increase the ability of the industry to improve cattle to better meet consumer expectations.

Numerous genome-wide association studies (GWAS) have been performed in different *Bos Taurus* (Gutiérrez-Gil et al., 2008; Esmailizadeh et al., 2011; McClure et al., 2012; Allais et al., 2014; Xia et al., 2016), *Bos Indicus* (Tizioto et al., 2013; Magalhães et al., 2016) or crossbred beef cattle breeds (Bolormaa et al., 2011; Lu et al., 2013; Hulsman Hanna et al., 2014), and with different phenotypes describing meat quality, from carcass characteristics to specific measures of eating satisfaction. These studies contribute to our present understanding of the genetic regulation for many of these traits but they also highlight some of the challenges and limitations associated with GWA studies. Many chromosomal regions identified are unique to the specific population in which they were discovered and were not replicated in other studies. More importantly, very few functional mutations have been identified and most of the genetic variation controlling these traits remains unknown. Recently, new methodology has been developed in an effort to address this limitation and allow for a better understanding of the genetic architecture of complex traits through a gene network analysis (Fortes et al., 2010; Reverter and Fortes, 2013).

The first objective of this study was to carry out GWAS to identify chromosomal regions associated with each of the different components of meat quality. The second objective was to use the Association Weight Matrices (AWM) and Partial Correlation and Information Theory (PCIT) to explore the functional mechanisms underlying GWAS associations for meat quality traits in Angus cattle to explore the biological mechanism by which GWAS-identified genomic variants give rise to phenotypic differences in eating quality.

## Materials and methods

### Animals and sample collection

The Iowa State University and Oklahoma State University Institutional Review Boards approved the experimental protocols used in this study. A total of 2,110 Angus-sired animals comprising bulls (*n* = 500), steers (*n* = 1,210), and heifers (*n* = 400) representing 155 sires were used in this study. All cattle were finished on concentrate diets in Iowa (*n* = 994), California (*n* = 345), Colorado (*n* = 352), or Texas (*n* = 419). Animals with an average age of 457 ± 46 days were harvested at commercial facilities. Details on production characteristics, meat sample collection, and preparation have been previously reported (Garmyn et al., 2011). Two 1.27-cm steaks from the longissimus muscle were trimmed of external fat and connective tissue and were analyzed for fatty acid and nutrient composition at Iowa State University (Ames, IA), using methods previously described (Garmyn et al., 2011; Mateescu et al., 2012) and for WBSF and sensory analyses at Oklahoma State University Food and Agricultural Products Center (Stillwater, OK) (Mateescu et al., 2015). Four carcass phenotypes: hot carcass weight (HCW), percentage kidney pelvic and heart fat (KPH), ribeye area (REA), and fat thickness (FAT); five meat quality phenotypes: marbling score (MS), IMFC, WBSF, sensory panel tenderness (TEND), sensory panel juiciness (JUIC); seven mineral concentrations: calcium, iron, magnesium, phosphorus, potassium, sodium, and zinc; four peptides: anserine, carnosine, creatine, and creatinine; and three groups of fatty acids: saturated (SFA), monounsaturated (MUFA), and polyunsaturated (PUFA) were used in this study. A description of the 23 traits along with a summary of descriptive statistics for this population is in Supplementary Table 1.

### Genome-wide association study (GWAS) of meat quality phenotypes

Genomic DNA extracted from the meat sample was genotyped with the Bovine SNP50 Infinium II BeadChip (Illumina, San Diego, CA). Those SNP with significant deviations from Hardy–Weinberg equilibrium at a significance level *P* < 0.0001 were removed prior to association analysis. Additionally, we used quality control filters for minor allele frequency (5%) and call rate for sample and SNP (95%). After quality control, 40,875 SNP were left and included in subsequent analyses. All GWAS were performed using the single-locus mixed linear model procedure implemented in Golden Helix SVS v8.4.4 software (Golden Helix Inc., Bozeman, MT, USA). The efficient mixed model association (EMMAX) approach in combination with a genomic relationship matrix was used to directly estimate the genetic and residual variance components $\sigma_{\text{g}}^{2}$ and $\sigma_{\text{e}}^{2}$ and the proportion of variance explained by the effects of significant SNP (Kang et al., 2010; Segura et al., 2012). In matrix notation, the basic model equation was:

$$\begin{array}{r} \text{Y}=\text{X}\beta +\text{g}+\text{e} \end{array}$$

Where Y is a vector of phenotypes for each of the meat quality traits measured on all the animals, β is the effect size of fixed effects (contemporary groups), g ~ N(0, $\sigma_{\text{a}}^{2}$K) is a random effect and e ~ N(0, $\sigma_{\text{e}}^{2}$I), where K is the genomic relationship matrix among animals.

Contemporary groups were defined based on gender at harvest (bull, steer, or heifer), finishing location (California, Colorado, Iowa, Texas), and harvest date, which resulted in a total of 33 groups. Contemporary groups were fit as fixed class effects in all genomic analyses. Pseudo-heritability was estimated as h^2^ = $\sigma_{\text{a}}^{2}$/($\sigma_{\text{e}}^{2}$ + $\sigma_{\text{e}}^{2}$) based on the estimates of the variance parameters (Kang et al., 2010). The *p*-values and additive genetic values for each SNP were obtained for each phenotype and these were used to construct the association weight matrix (AWM; Reverter and Fortes, 2013).

### Association weight matrix

The AWM approach (Reverter and Fortes, 2013) was used to interpret the results from GWAS. The WBSF was selected as the key phenotype to describe the complex of traits related to tenderness and meat quality. An initial set of 1,842 SNP with largest estimated additive effects for WBSF were selected based on their raw *P* < 0.05. A less stringent level at this stage is recommended to allow for a proper integration of potentially important regulators across multiple traits. One advantage of the AWM/PCIT methodology is the ability to include SNP with relatively small effects which do not reach genome-wide statistical significance but are potentially linked to elements controlling the trait of interest. It is well-recognized that many elements with minor effects are usually not able to reach significance at the genome level, but they will be uncovered through a gene network when multiple correlated traits are used in the analysis (Fortes et al., 2010). The average number of other phenotypes associated with these SNP at a *P* < 0.05 was calculated and 1,318 SNP associated with at least two phenotypes were included in the AWM. To build the AWM, a vector of posterior mean estimates of 1,318 SNP effects from WBSF was enhanced with the vectors of effects of all the other 22 phenotypes. This 1,318 × 22 matrix of posterior mean estimates of SNP effects was used as the input for PCIT to detect similar effects for any SNP across multiple phenotypes. All SNP pairs within the matrix were tested for association with at least one other SNP in order to establish network connections. SNP pairs without a significant partial correlation to at least one other SNP were removed from the dataset to discard them from subsequent network association analysis since they would appear isolated.

Networks of SNP showing common effects across multiple quality traits were constructed based on the computed correlations among SNP. Correlation between SNP pairs with a non-zero partial correlation to another SNP were input into Cytoscape 3.5.1 (Shannon et al., 2003) software to create gene network clusters using the MCODE plugin (Bader and Hogue, 2003; Saito et al., 2012). Networks were scored and ranked by the MCODE algorithm as network density times the number of nodes. The MCODE algorithm defines network density as the number of edges in a network divided by the theoretical maximum number of edges in the network. The SNP that comprised the network were annotated with the Variant Effect Predictor (VEP) using Bovine UMD 3.1 annotations (McLaren et al., 2010).

### Gene ontology enrichment analysis and visualization

DAVID v6.7 Functional Annotation Tool (Huang et al., 2009) was used for gene ontology (GO) enrichment in order to detect enriched biological terms associated with genomic regions and gene networks identified in the analysis. The GO term enrichment and clustering was performed on all annotated genes associated with the quality traits. Functional grouping based on kappa score and visualization in a functionally grouped network was performed using the ClueGO (Bindea et al., 2009) plug-in in Cytoscape. A *P* < 0.05 and kappa coefficient > 0.3 were considered as threshold values.

## Results and discussion

### Meat quality genome-wide association study

Summary statistics for carcass quality, meat quality, mineral content, fatty acid composition, and peptide content phenotypes are presented in Table 1 along with heritability estimates for each trait and general GWAS information. Complete GWAS results for all 23 individual meat quality traits are presented in Supplementary Table 1. The GWAS for our main meat quality trait, WBSF, resulted in 1,878 SNP associated with this trait at *P* < 0.05, of which there were 383 SNP at *P* < 0.01 and 56 SNP at *P* < 0.001. A list with detailed information on the top 35 markers (*P* < 0.00005) associated with WBSF is in Table 2, and additional information in Supplementary Table 2. The number of significant SNP was similar across all 23 traits and ranged from 1,729 to 1,971 at *P* < 0.05, from 331 to 428 at *P* < 0.01, and 25 to 112 at *P* < 0.001. There were 68 SNP significantly associated with 10 or more traits at *P* < 0.05 and 7 SNP significantly associated with 15 or more traits (Table 3, additional information in Supplementary Table 3). The most significant regions for WBSF were identified, in order of significance, on BTA29, 20, 10, 7, 3, and 4. Most of these chromosomal regions harbor potential candidate genes for tenderness that have been identified in other studies in several cattle breeds. Among these, *CAST* (on BTA7) and *CAPN1* (on BTA29) have been consistently identified and have a role in muscle proteolysis during meat aging (Smith et al., 2000). In fact, 13 out of the 56 SNP significant for WBSF at *P* < 0.001 were located in a 3 cM region around CAPN1 (three SNP directly in CAPN1) and four of the 56 SNP were located around CAST.

**Table 1:** Phenotypic data and GWAS information for traits describing the meat quality complex.

**Trait**	***N***	**Mean**	**StDev**	**Min**	**Max**	***h*^2^**	***p* < 0.05**	***p* < 0.01**	***p* < 0.001**
**CARCASS QUALITY**									
HCW, kg	2,110	332.67	32.36	222.26	453.14	0.26	1,913	352	44
Fat Thickness, cm	2,110	1.25	0.47	0.31	3.15	0.67	1,942	390	45
KPH, %	2,110	2.08	0.40	1	3.5	0.23	1,844	373	48
**MEAT QUALITY**									
LM area, cm^2^	2,110	81.21	7.98	55.48	118.06	0.39	1,971	386	36
Tenderness	1,591	5.80	0.59	3	7.375	0.33	1,889	400	52
WBSF, kg	2,076	3.53	0.77	1.491	8.467	0.38	1,842	383	56
Juiciness	1,591	5.00	0.50	3.375	6.375	0.22	1,943	353	31
Marbling Score	2,109	5.96	1.04	3	9.8	0.40	1,949	389	41
IMFC, %	2,110	5.67	2.22	0.23	26.4	0.40	1,878	388	61
**MINERAL CONTENT**									
Ca, μg/g	2,099	38.87	20.88	2.01	218.54	0.17	1,969	382	42
Fe, μg/g	2,087	14.44	3.03	5.2	27.43	0.59	1,956	425	53
K, μg/g	2,054	3433.54	494.27	1306.16	4895.9	0.43	1,775	357	52
Mg, μg/g	2,102	254.54	43.06	156.39	440.74	0.65	1,748	352	46
Na, μg/g	2,101	489.44	92.92	213.13	855.05	0.56	1,922	423	62
P, μg/g	2,102	1965.55	286.39	0.82	3163.15	0.46	1,786	339	42
Zn, μg/g	2,090	38.96	7.90	8.55	85.81	0.30	1,878	385	44
**FATTY ACID COMPOSITION**									
SFA, %	2,010	45.29	2.38	35.41	55.88	0.56	1,851	428	73
MUFA,%	2,010	49.05	2.79	35.86	57.68	0.39	1,867	399	62
PUFA, %	2,010	5.67	1.85	1.17	18.21	0.28	1,934	399	28
**PEPTIDE CONTENT**									
Anserine	1,995	0.67	0.14	0.05	1.22	0.64	1,747	423	112
Carnosine	1,993	3.72	0.47	0.75	5.72	0.48	1,885	390	69
Creatine	1,710	5.26	0.53	1.89	6.86	0.47	1,833	398	57
Creatinine	2,007	0.21	0.11	0.03	0.55	0.59	1,729	331	48

*For each trait number of animals (N), average (Mean), standard deviation (StDev), minimum (Min) and maximum (Max) values are presented along with an estimate of the pseudo-heritability (h^2^), and number of SNP with p < 0.05, 0.01 or 0.001 from the GWAS on each component trait*.

**Table 2:** List of the top 35 markers (*P* < 0.00005) associated with Warner-Bratzler Shear force (WBSF, kg).

**Marker Name**	**BTA**	**Position (bp)**	***p*-value**	**Effect**
rs110680201	2	120,073,875	2.15 × 10^−4^	0.0012186
rs110822981	3	13,704,030	2.82 × 10^−5^	0.0020604
rs110355365	3	42,339,927	1.00 × 10^−4^	0.0020308
rs109050625	4	101,790,675	5.90 × 10^−5^	−0.0022019
rs109804679	7	98,498,047	1.96 × 10^−4^	0.0017956
rs109677393	7	98,534,197	1.63 × 10^−5^	0.0020727
rs41657604	10	102,707,947	7.28 × 10^−6^	0.0024819
rs109487930	12	28,022,872	1.78 × 10^−4^	0.0020716
rs110752731	15	3,600,480	2.89 × 10^−4^	0.001649
rs110584426	15	30,573,210	4.72 × 10^−4^	0.0014741
rs29026935	15	32,783,311	4.63 × 10^−4^	0.0018346
rs41950387	20	57,373,160	8.04 × 10^−6^	0.0018595
rs41997980	22	13,400,771	1.28 × 10^−4^	−0.0014818
rs41603459	22	30,010,174	1.65 × 10^−4^	−0.0020498
rs41659707	24	13,810,452	1.75 × 10^−4^	0.0017118
rs29019820	24	36,077,466	4.78 × 10^−4^	0.0013192
rs41608068	29	1,573,172	4.55 × 10^−4^	0.0020577
rs109830547	29	4,533,981	3.54 × 10^−4^	0.0011529
rs109710777	29	37,152,168	2.67 × 10^−4^	−0.0014565
rs109814977	29	43,525,624	1.31 × 10^−5^	−0.0015407
rs110770404	29	43,611,640	3.11 × 10^−4^	0.0019914
rs17872000	29	44,069,063	7.91 × 10^−7^	−0.0024809
rs17871058	29	44,085,769	2.90 × 10^−4^	0.0019333
rs17872050	29	44,087,629	1.75 × 10^−4^	0.0020251
rs110294629	29	44,325,408	9.54 × 10^−6^	−0.0021288
rs42191092	29	44,546,564	1.63 × 10^−4^	0.0020548
rs110174152	29	44,585,782	4.77 × 10^−4^	0.0019724
rs800857481	29	46,646,575	1.85 × 10^−4^	0.0018152
rs42199297	29	46,703,510	3.02 × 10^−4^	0.0020764
rs42194740	29	46,732,932	3.31 × 10^−4^	0.0020627
rs42845824	29	46,999,731	1.52 × 10^−4^	0.0016505
rs29010111	X	20,453,664	4.52 × 10^−4^	0.0013076
rs41609600	X	62,311,454	3.11 × 10^−4^	−0.0027251
rs41626493	X	97,403,554	2.99 × 10^−4^	−0.0024891
rs41628805	X	141,578,318	4.32 × 10^−4^	0.0025361

*Chromosome (BTA), position on the chromosome (bp), p-value and allele substitution effect (Effect)*.

**Table 3:** Top 30 markers significantly associated with 10 or more meat quality traits at *P* < 0.05.

**Marker**	**BTA**	**Position (bp)**	**No Traits**	**Consequence**
rs109734539	1	68,937,163	10	Upstream gene variant
rs109251210	1	156,366,103	11	Intergenic variant
rs108949614	3	55,074,485	10	Intron variant
rs109507539	3	96,660,603	10	3 prime UTR variant
rs109977837	3	110,272,602	11	Intron variant
rs43157198	4	41,128,696	11	Intergenic variant
rs41588698	4	59,710,881	11	Intergenic variant
rs42715455	6	6,955,308	15	Intron variant
rs110018485	7	22,524,899	12	Intron variant
rs41700602	7	36,884,206	11	Intergenic variant
rs109977037	7	90,900,133	11	Non coding transcript exon variant
rs109819349	7	91,836,262	15	Intergenic variant
rs41625563	7	91,903,228	15	Intergenic variant
rs110059753	7	92,033,645	17	Intergenic variant
rs41625576	7	93,289,032	11	Intergenic variant
rs109627006	7	93,396,872	12	Intergenic variant
rs110612774	8	64,208,930	11	Intergenic variant
rs109242304	9	11,526,739	11	Intergenic variant
rs108987903	11	45,175,551	11	Intergenic variant
rs110587871	14	13,081,432	11	Intergenic variant
rs41631415	14	57,631,331	11	Intergenic variant
rs109560127	15	56,782,573	22	Intergenic variant
rs110308812	19	56,533,680	14	Intron variant
rs29018751	20	37,297,072	11	Intron variant
rs41256507	21	39,470,288	11	Intergenic variant
rs41659707	24	13,810,452	11	Intergenic variant
rs109257502	26	25,253,444	19	Intron variant
rs109611741	26	41,414,375	13	Intergenic variant
rs29021718	27	2,378,910	14	Intergenic variant

*The location in bp (UMD3.1) of each marker and the chromosome (BTA), the number of traits significantly associated at P < 0.05) and the marker consequence*.

### Meat quality gene networks

Among the 1,842 SNP significant (*P* < 0.05) for WBSF, there were 839 SNP associated with at least two other phenotypes. Some 772 SNP were found to be located within a gene (*n* = 712) or within 2.5 kbp from a gene (*n* = 60) and therefore were used to form the AWM. A total of 688 annotated genes were found associated with at least one other gene and had significant direct and partial correlations. This correlation matrix generated a gene network consisting of 688 genes (nodes) and 99,568 gene relationships (edges). The Cytoscape MCODE plugin colocalized these SNP into 17 separate networks and detailed information on the top five networks is in Table 4. Nodes with no gene or feature annotation were removed for visual simplicity from the figures. The significance of each node is indicated by its location within the network, and the distance from the center indicates the total number of connections and importance to the phenotype. A direct correlation detected through the PCIT analysis is represented as a connection or edge in the network. The highest scoring network contained 324 nodes and 53,424 edges, or connections. The clusters of genes represent scored networks derived through the PCIT analysis, and theoretically these clusters of genes function as molecular complexes controlling the specified phenotype.

**Table 4:** MCODE results derived from network clustering with PCIT.

**Network**	**Score**	**Nodes**	**Edges**
1	280.75	324	53,424
2	41.19	93	1,987
3	19.33	49	524
4	10.76	30	156
5	8.94	18	76

*Top five network scores from MCODE plugin for meat quality traits. Network score represents density of nodes and edges in each network*.

There are numerous candidate genes within these networks involved in metabolic and cellular processes that have possible impacts on meat quality traits. The genes CAPN1 and CAST, are well-known candidate genes for tenderness and meat quality traits (Goll et al., 1992; Geesink and Koohmaraie, 1999; Page et al., 2002; Casas et al., 2014), and are identified as major nodes in two subnetworks. There are many other candidate genes with a high network score indicating a high number of direct and indirect correlations with supporting evidence in the literature for their relationship to muscle growth and metabolism, calcium metabolism, adipogenesis, extracellular matrix protein interactions, and regulation. The gene MYOM1 (myomesin 1) is expressed in muscle cells and contributes to the three-dimensional conformation stability of the thick filament (Moreno-Sánchez et al., 2010; Picard et al., 2015). The CALCOCO1 gene (Calcium Binding and Coiled-Coil Domain 1) was shown to provide a link between cellular metabolism (phosphate and glucose metabolism), protein synthesis and degradation, calcium signaling and cell growth (Yang et al., 2006). The gene ALDOA (Aldolase A), that may encode a scaffolding protein, plays a key role in glycolysis and gluconeogenesis (Hocquette and Gigli, 2005; D'Alessandro and Zolla, 2013; Gobert et al., 2014). Among the 3 isozymes (A, B, and C), Aldolase A is present in the developing embryo and it is found in greater quantities in the skeletal adult muscle where it accumulates around the M line and within the I band, localizing with FBP2 on both sides of the Z line in the absence of calcium. ADAMTS15 encodes a member of the ADAMTS (a disintegrin and metalloproteinase with thrombospondin motifs) protein family (Stanton et al., 2011). The encoded preproprotein is proteolytically processed to generate the mature enzyme, which may play a role in versican processing during skeletal muscle development (De Jager et al., 2013; Mudadu et al., 2016). KLHL2 is a component of an ubiquitin-protein ligase complex that mediates the ubiquitination of target proteins, which most often leads to their proteasomal degradation and plays a role in the actin cytoskeleton reorganization. The CRTAC1 gene encodes a glycosylated extracellular matrix protein located in the interterritorial matrix of articular deep zone cartilage and the protein may be involved in cell-cell or cell-matrix interactions (Anjos et al., 2017). Overall, these examples provide strong evidence that the network methodology used in this study allows the co-localization of biologically relevant genes with a close relationship to different aspects of meat quality variation. TNS4 (tensin 4) encodes an actin binding protein involved in cell migration, cartilage development and in connecting signal transduction pathways to the cytoskeleton (Van de Werken et al., 1993; Lo, 2004). The gene encoded by COL27A1 is a member of the fibrillar collagen family, and plays a role during the calcification of cartilage and the transition of cartilage to bone (Pace et al., 2003).

### Gene ontology term enrichment analysis

**Figure 1:**
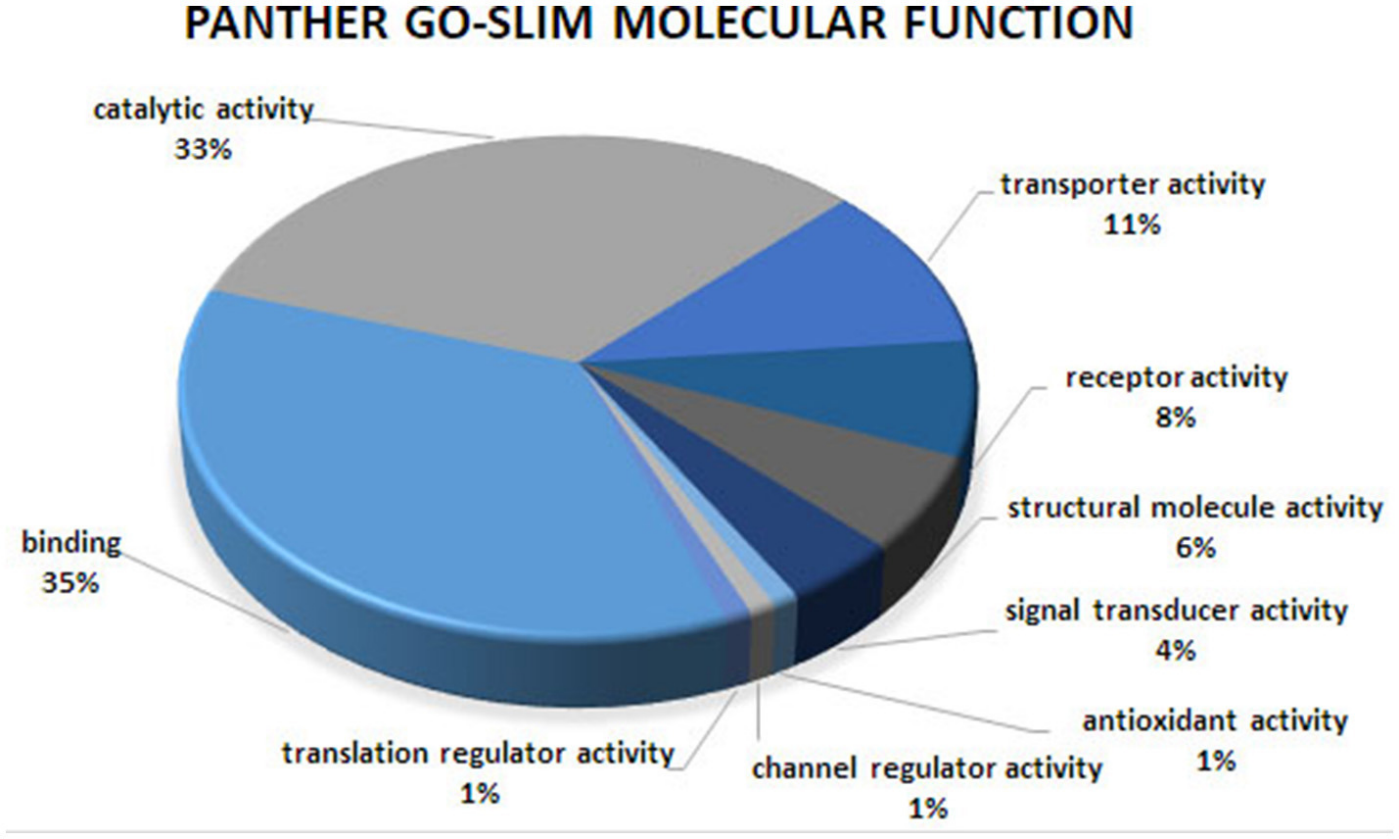
Molecular function analysis of the co-association network for meat quality complex. The PANTHER overrepresentation test grouped 609 annotated genes into 9 molecular function classes.

**Figure 2:**
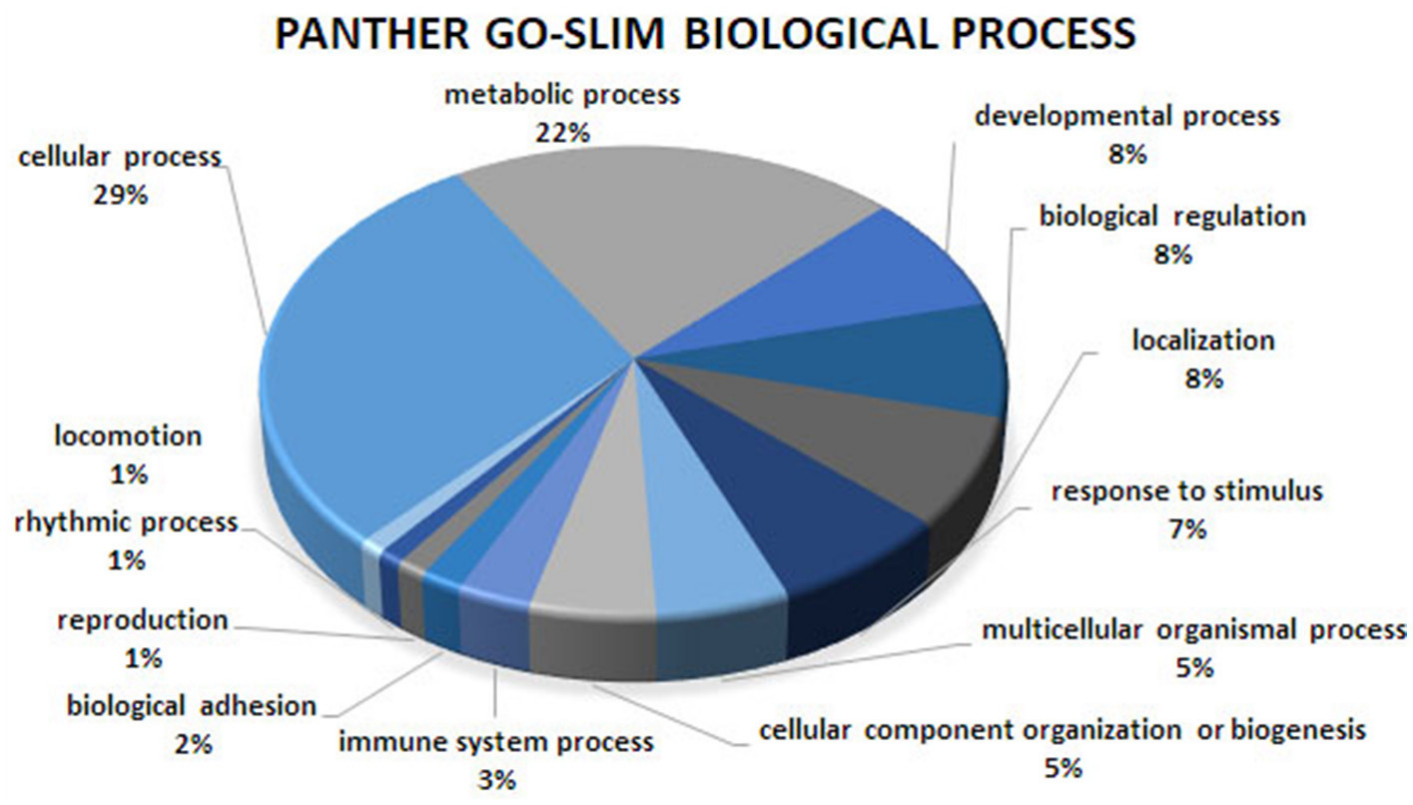
Biological process analysis of the co-association network for meat quality complex. The PANTHER overrepresentation test grouped 609 annotated genes into 13 biological processes.

**Figure 3:**
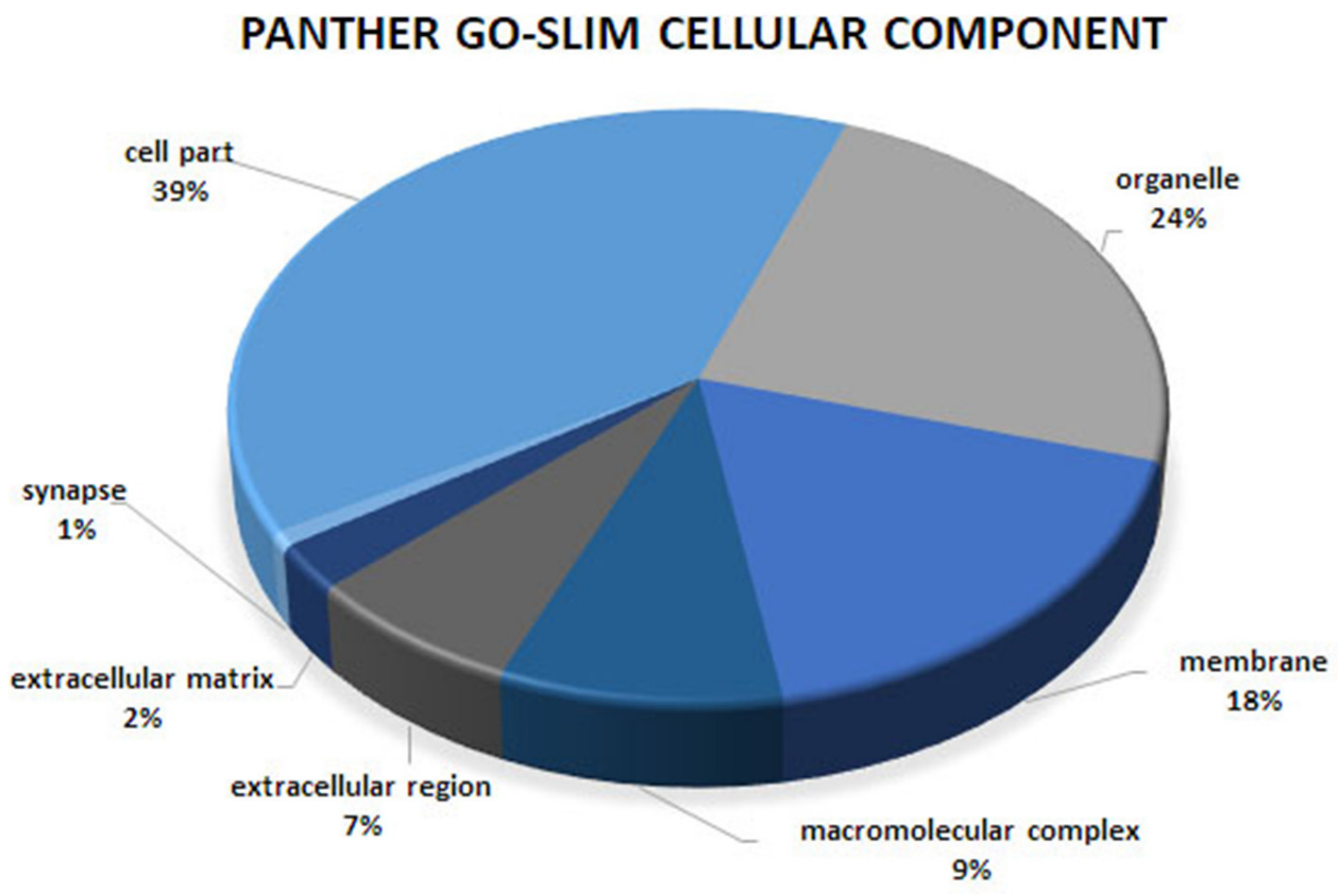
Cellular component analysis of the co-association network for meat quality complex. The PANTHER overrepresentation test grouped 609 annotated genes into 7 cellular components.

Gene ontology and pathway enrichment analyses were carried out to gain insight into the predicted gene networks using PANTHER Overrepresentation Test and DAVID Functional Classification Clustering tools. The PANTHER classifications are presented according to molecular function, biological process, and cellular component in Figures 1–3. Significant results for the DAVID Functional Annotation Clustering results for the gene networks are in Table 5. An enrichment score of 1.3 was used as a significance threshold for DAVID Functional Annotation Clusters while a *P* < 0.05 was used to designate the functional annotation chart GO terms as significantly enriched (Huang et al., 2009). The false discovery rate (FDR) included in the Functional Annotation Chart can be used to determine the importance of terms considered significant through the *P*-value statistic.

**Table 5:** DAVID Functional Annotation Clustering for the 688 annotated genes in the gene network for meat quality complex.

**Category**	**Term**	**Count**	**%**	***P*-value**	**FE**	**FDR**
**Annotation Cluster 1**	**Enrichment Score: 2.82**					
UP_ KEYWORDS	Ion channel	20	3.21	< 0.01	2.68	0.24
UP_ KEYWORDS	Ion transport	27	4.33	< 0.01	2.11	0.65
UP_ KEYWORDS	Transport	48	7.70	0.03	1.33	37.81
**Annotation Cluster 2**	**Enrichment Score: 1.76**					
INTERPRO	IPR005821:Ion transport domain	12	1.93	< 0.01	3.48	1.04
GOTERM_ BP_DIRECT	GO:0086010~membrane depolarization during action potential	4	0.64	0.07	4.19	69.79
INTERPRO	IPR027359:Voltage-dependent potassium channel, four helix bundle domain	5	0.80	0.1	2.69	85.67
**Annotation Cluster 3**	**Enrichment Score: 1.71**					
UP_ KEYWORDS	EGF-like domain	12	1.93	< 0.01	2.65	7.32
INTERPRO	IPR018097:EGF-like calcium-binding, conserved site	9	1.44	< 0.01	3.06	13.55
SMART	SM00181:EGF	14	2.25	0.01	2.22	12.87
INTERPRO	IPR001881:EGF-like calcium-binding	10	1.61	0.01	2.66	18.66
INTERPRO	IPR000742:Epidermal growth factor-like domain	14	2.25	0.01	2.10	23.32
SMART	SM00179:EGF_CA	10	1.61	0.03	2.22	37.71
INTERPRO	IPR013032:EGF-like, conserved site	11	1.77	0.07	1.88	68.06
INTERPRO	IPR000152:EGF-type aspartate/asparagine hydroxylation site	7	1.12	0.08	2.30	75.32
**Annotation Cluster 4**	**Enrichment Score: 1.63**					
KEGG_ PATHWAY	bta04724:Glutamatergic synapse	12	1.93	< 0.01	3.69	0.47
INTERPRO	IPR001828:Extracellular ligand-binding receptor	6	0.96	< 0.01	5.22	8.19
INTERPRO	IPR001508:NMDA receptor	4	0.64	0.01	6.96	25.56
INTERPRO	IPR001320:Ionotropic glutamate receptor	4	0.64	0.01	6.96	25.56
UP_ KEYWORDS	Ligand-gated ion channel	6	0.96	0.02	3.53	29.87
UP_ KEYWORDS	Postsynaptic cell membrane	7	1.12	0.02	3.04	30.15
SMART	SM00079:PBPe	4	0.64	0.03	5.81	32.34
GOTERM_ CC_DIRECT	GO:0045211~postsynaptic membrane	8	1.28	0.05	2.31	56.25
INTERPRO	IPR019594:Glutamate receptor, L-glutamate/glycine-binding	3	0.48	0.09	5.55	81.47
GOTERM_ MF_DIRECT	GO:0005234~extracellular-glutamate-gated ion channel activity	3	0.48	0.1	5.42	79.59
SMART	SM00918:SM00918	3	0.48	0.1	4.63	85.18

*Statistics associated with GO terms include significance of enrichment or EASE score (P-value), fold enrichment (FE), and false discovery rate (FDR)*.

Overrepresented terms for GO-Slim Molecular Function in the network included “Transmembrane Transporter Activity,” “Ligand-Gated Ion Channel,” “Extracellular Matrix Protein,” “Transporter,” “Nucleic Acid Binding,” and “RNA binding protein” for PANTHER Protein Class; “Ion Binding,” “G-Protein Coupled Receptor Activity,” and “Receptor Activity” for GO Molecular Function. Functional annotation analyses revealed an enrichment for “Ion Binding” (*P* = 1.98 × 10^−9^), “Protein Binding” (*P* = 8.48 × 10^−8^), “Catalytic Activity” (*P* = 4.44 × 10^−5^), “Metal Ion Transmembrane Transporter Activity” (*P* = 5.89 × 10^−5^), “Enzyme Binding” (*P* = 8.14 × 10^−5^), “Transmembrane Transporter Activity” (*P* = 3.4 × 10^−4^), and “Gated Channel Activity” (*P* = 6.73 × 10^−3^). We found 21 over-represented pathways including “Angiogenesis,” “Inflammation mediated by chemokine and cytokine signaling pathway,” “Ionotropic glutamate receptor pathway,” “TGF-beta signaling pathway,” “Apoptosis signaling pathway.” Many of these pathways have been previously reported as important biological pathways involved in meat quality or tenderness in beef cattle (Guillemin et al., 2012; Mudadu et al., 2016; Ramayo-Caldas et al., 2016). An investigation of genes overrepresented in the 21 pathways revealed 26 genes common to at least 10 pathways. Six genes namely KCNIP4, GAS6, KCNH2, RYR1, ATP2B1, and HCN1 were found in common between at least 14 pathways. It is interesting to note that a majority of the common genes that we detected are involved in calcium-related processes: calcium ion binding, calcium channel, calcium-transporting ATPase, and calcium channel regulator. This is not surprising given the role of calcium and potassium in meat tenderness through their involvement in the proteolytic system responsible for postmortem tenderization and muscle contraction.

**Figure 4:**
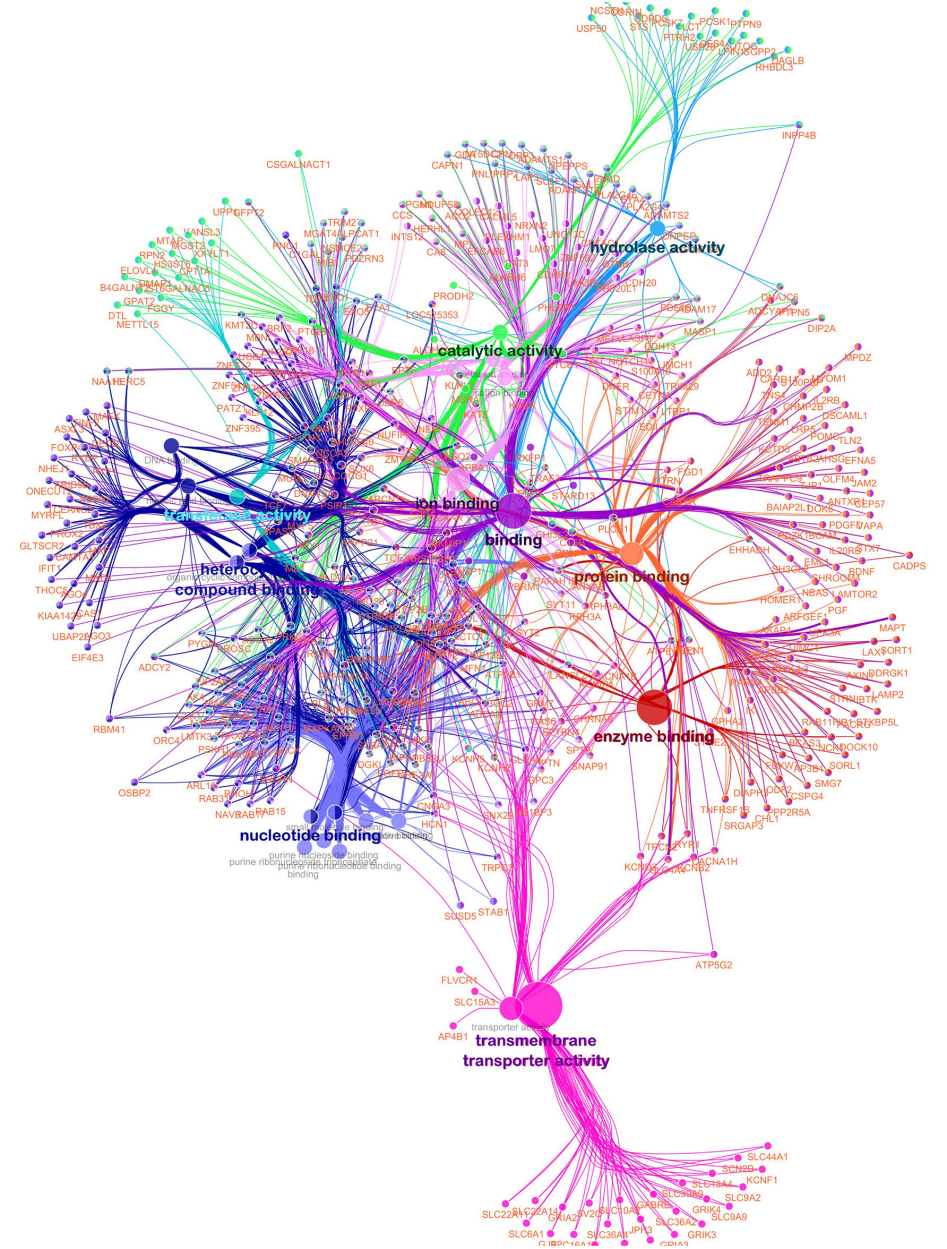
Functionally grouped network for meat quality complex in Angus cattle. Nodes represent functional terms linked based on their kappa score level (>0.3) with only the most significant term per group shown as a label. The node size represents the enrichment significance of the term. Only genes in common between two or more GO terms are used.

A functionally grouped annotation network (Figure 4) was developed based on 576 unique and annotated genes from the AWM/PCIT analysis and the network was visualized using the ClueGO plug-in for Cytoscape. Only 544 genes were recognized by ClueGO, 454 (83.46%) were functionally annotated in the “Molecular Function” ontology, and 442 (81.25%) were associated with representative terms and pathways after applying the selection criteria. Twenty-two GO terms were significantly represented in this network. The most representative term was “Binding” with 358 genes and 3.58% associated genes, followed by “Ion Binding,” “Protein binding,” Catalytic activity,” and “Organic Cyclic compound binding.” Higher connectivity between GO terms with similar molecular function are to be expected, but a high priority in terms of future research will be placed on genes common between several different GO terms as these might point toward key regulator genes with higher impact on the meat quality complex. The type of analyses used in this study and aimed at dissecting and understanding the gene networks and their contribution to the phenotypic expression of complex traits is highly dependent on the level of annotation of the respective genome but will further our general knowledge of gene function.

The gene network technique employed in this work advances the genomic analysis of complex traits beyond the simple marker association analysis by allowing the inclusion of markers which initially are not able to reach a very stringent genome-wide significance status. These markers and the genomic regions they represent could be legitimate markers and regions explaining a small portion of the variation in these complex traits, but they do not have large enough effects in order to reach significance. The danger of a false positive is overcome through the gene/network enrichment analysis where a true false positive gene would most likely be eliminated while genes with a real but small effect on the trait will be validate through their biological role in a specific pathway contributing to trait of interest. However, it is important that these results are validated through additional functional analyses at the gene expression or proteomics level.

## Conclusion

Traits including four carcass measures, five meat quality phenotypes, seven mineral concentrations, and four peptide concentrations were used in GWAS to populate a gene network analysis using the methodology of AWM/PCIT. An analysis of genomic regions that affect different aspects of meat quality highlighted genes overrepresented in molecular functions related to calcium and other ion binding and regulation, catalytic and transporter activity, and nucleic acid and RNA binding. Several genes were found in a majority of enriched pathways suggesting possible key regulatory roles for these genes. This also provides evidence for the interconnections between the individual pathways and sheds some light on how these different pathways control the meat quality phenotype. The combination of GWAS results with PCIT and network visualization represents a powerful methodology for identifying novel candidate genes of interest for complex traits influenced by multiple component phenotypes. This methodology allows for a dissection of the biological mechanisms and gene networks that lead to these complex phenotypes.

## Author contributions

RM conceived and conducted the analysis and drafted the manuscript. JR assisted with the analysis and manuscript. DG assisted with the analysis and manuscript.

### Conflict of interest statement

The authors declare that the research was conducted in the absence of any commercial or financial relationships that could be construed as a potential conflict of interest. This research was supported by Pfizer Animal Genetics. The funder was not involved in the study design or collection, analysis, or interpretation of the data.

## References

[B1] Allais S. Levéziel H. Hocquette J. F. Rousset S. Denoyelle C. Journaux L.. (2014). Fine mapping of quantitative trait loci underlying sensory meat quality traits in three French beef cattle breeds. J. Anim. Sci. 92, 4329–4341. 10.2527/jas.2014-7868 25149327

[B2] Anjos L. Morgado I. Guerreiro M. Cardoso J. C. R. Melo E. P. Power D. M. (2017). Cartilage acidic protein 1, a new member of the beta-propeller protein family with amyloid propensity. Proteins Struct. Funct. Bioinform. 85, 242–255. 10.1002/prot.25210 27862299

[B3] Bader G. D. Hogue C. W. V. (2003). An automated method for finding molecular complexes in large protein interaction networks. BMC Bioinform. 4:2. 10.1186/1471-2105-4-2 12525261 PMC149346

[B4] Bindea G. Mlecnik B. Hackl H. Charoentong P. Tosolini M. Kirilovsky A.. (2009). ClueGO: a Cytoscape plug-in to decipher functionally grouped gene ontology and pathway annotation networks. Bioinformatics 25, 1091–1093. 10.1093/bioinformatics/btp101 19237447 PMC2666812

[B5] Bolormaa S. Porto Neto L. R. Zhang Y. D. Bunch R. J. Harrison B. E. Goddard M. E.. (2011). A genome-wide association study of meat and carcass traits in australian cattle. J. Anim. Sci. 89, 2297–2309. 10.2527/jas.2010-3138 21421834

[B6] Casas E. Duan Q. Schneider M. J. Shackelford S. D. Wheeler T. L. Cundiff L. V.. (2014). Polymorphisms in calpastatin and mu-calpain genes are associated with beef iron content. Anim. Genet. 45, 283–284. 10.1111/age.12108 24303986

[B7] D'Alessandro A. Zolla L. (2013). Meat science: From proteomics to integrated omics towards system biology. J. Proteomics 78, 558–577. 10.1016/j.jprot.2012.10.023 23137709

[B8] De Jager N. Hudson N. J. Reverter A. Barnard R. Cafe L. M. Greenwood P. L.. (2013). Gene expression phenotypes for lipid metabolism and intramuscular fat in skeletal muscle of cattle. J. Anim. Sci. 91, 1112–1128. 10.2527/jas.2012-5409 23296809

[B9] Fortes M. R. S. Reverter A. Zhang Y. Collis E. Nagaraj S. H. Jonsson N. N.. (2010). Association weight matrix for the genetic dissection of puberty in beef cattle. Proc. Natl. Acad. Sci. U.S.A. 107, 13642–13647. 10.1073/pnas.1002044107 20643938 PMC2922254

[B10] Garmyn A. J. Hilton G. G. Mateescu R. G. Morgan J. B. Reecy J. M. Tait J. G.. (2011). Estimation of relationships between mineral concentration and fatty acid composition of longissimus muscle and beef palatability traits. J. Anim. Sci. 89, 2849–2858. 10.2527/jas.2010-3497 21512113

[B11] Geesink G. H. Koohmaraie M. (1999). Effect of calpastatin on degradation of myofibrillar proteins by mu-calpain under postmortem conditions. J. Anim. Sci. 77, 2685–2692. 10.2527/1999.77102685x 10521028

[B12] Gobert M. Sayd T. Gatellier P. Santé-Lhoutellier V. (2014). Application to proteomics to understand and modify meat quality. Meat Sci. 98, 539–543. 10.1016/j.meatsci.2014.06.035 25041652

[B13] Goll D. E. Thompson V. F. Taylor R. G. Christiansen J. A. (1992). Role of the calpain system in muscle growth. Biochimie 74, 225–237. 10.1016/0300-9084(92)90121-T 1610936

[B14] Guillemin N. P. Jurie C. Renand G. Hocquette J.-F. Micol D. Lepetit J. (2012). Different phenotypic and proteomic markers explain variability of beef tenderness across muscles. Int. J. Biol. 4, 26–38. 10.5539/ijb.v4n2p26

[B15] Gutiérrez-Gil B. Wiener P. Nute G. R. Burton D. Gill J. L. Wood J. D.. (2008). Detection of quantitative trait loci for meat quality traits in cattle. Anim. Genet. 39, 51–61. 10.1111/j.1365-2052.2007.01682.x 18254735

[B16] Hocquette J. Gigli S. (2005). Indicators of Milk and Beef Quality. Available online at: http://books.google.com/books?hl=en&lr=&id=HSK1SwrWq7sC&oi=fnd&pg=PA13&dq=Indicators+of+milk+and+beef+quality&ots=Ocel_wMYyS&sig=UhsTyWiioJ0gn0xOFc6O_LnvIm4 (Accessed March 12, 2013). 10.3920/978-90-8686-537-6

[B17] Huang D. W. Sherman B. T. Lempicki R. A. (2009). Systematic and integrative analysis of large gene lists using DAVID bioinformatics resources. Nat. Protoc. 4, 44–57. 10.1038/nprot.2008.211 19131956

[B18] Hulsman Hanna L. L. Garrick D. J. Gill C. A. Herring A. D. Riggs P. K. Miller R. K. (2014). Genome-wide association study of temperament and tenderness using different Bayesian approaches in a Nellore-Angus crossbred population. Livest. Sci. 161, 17–27. 10.1016/j.livsci.2013.12.012

[B19] Igo J. L. Vanoverbeke D. L. Woerner D. R. Tatum J. D. Pendell D. L. Vedral L. L.. (2013). Phase I of The National Beef Quality Audit - 2011: quantifying willingness-to-pay, best worst scaling, and current status of quality characteristics in different beef industry marketing sectors. J. Anim. Sci. 91, 1907–1919. 10.2527/jas.2012-5815 23408805

[B20] Kang H. M. Sul J. H. Service S. K. Zaitlen N. A. Kong S.-Y. Freimer N. B.. (2010). Variance component model to account for sample structure in genome-wide association studies. Nat. Genet. 42, 348–354. 10.1038/ng.548 20208533 PMC3092069

[B21] Esmailizadeh A. K. Morris C. A. Cullen N. G. Kruk Z. A. Lines D. S. Hickey S. M.. (2011). Genetic mapping of quantitative trait loci for meat quality and muscle metabolic traits in cattle. Anim. Genet. 42, 592–599. 10.1111/j.1365-2052.2011.02197.x 22035000

[B22] Koohmaraie M. Kent M. P. Shackelford S. D. Veiseth E. Wheeler T. L. (2002). Meat tenderness and muscle growth: is there any relationship? Meat Sci. 62, 345–352. 10.1016/S0309-1740(02)00127-4 22061610

[B23] Lo S. H. (2004). Tensin. Int. J. Biochem. Cell Biol. 36, 31–34. 10.1016/S1357-2725(03)00171-7 14592531

[B24] Lu D. Sargolzaei M. Kelly M. Vander Voort G. Wang Z. Mandell I.. (2013). Genome-wide association analyses for carcass quality in crossbred beef cattle. BMC Genet. 14:80. 10.1186/1471-2156-14-80 24024930 PMC3827924

[B25] Magalhães A. F. B. de Camargo G. M. F. Fernandes G. A. Gordo D. G. M. Tonussi R. L. Costa R. B.. (2016). Genome-wide association study of meat quality traits in nellore cattle. PLoS ONE 11:e0157845. 10.1371/journal.pone.0157845 27359122 PMC4928802

[B26] Mateescu R. G. Garrick D. J. Garmyn A. J. Vanoverbeke D. L. Mafi G. G. Reecy J. M. (2015). Genetic parameters for sensory traits in longissimus muscle and their associations with tenderness, marbling score, and intramuscular fat in Angus cattle. J. Anim. Sci. 93, 21–27. 10.2527/jas.2014-8405 25412744

[B27] Mateescu R. G. Garmyn A. J. O'Neil M. A. Tait R. G. Abuzaid A. Mayes M. S.. (2012). Genetic parameters for carnitine, creatine, creatinine, carnosine, and anserine concentration in longissimus muscle and their association with palatability traits in angus cattle. J. Anim. Sci. 90, 4248–4255. 10.2527/jas.2011-5077 22952371

[B28] Mateescu R. G. Oltenacu P. A. Garmyn A. J. Mafi G. G. VanOverbeke D. L. (2016). Strategies to predict and improve eating quality of cooked beef using carcass and meat composition traits in Angus cattle. J. Anim. Sci. 94:2160. 10.2527/jas.2015-0216 27285712

[B29] McClure M. C. Ramey H. R. Rolf M. M. McKay S. D. Decker J. E. Chapple R. H.. (2012). Genome-wide association analysis for quantitative trait loci influencing Warner-Bratzler shear force in five taurine cattle breeds. Anim. Genet. 43, 662–673. 10.1111/j.1365-2052.2012.02323.x 22497286 PMC3506923

[B30] McLaren W. Pritchard B. Rios D. Chen Y. Flicek P. Cunningham F. (2010). Deriving the consequences of genomic variants with the Ensembl API and SNP Effect Predictor. Bioinformatics 26, 2069–2070. 10.1093/bioinformatics/btq330 20562413 PMC2916720

[B31] Moreno-Sánchez N. Rueda J. Carabaño M. J. Reverter A. McWilliam S. González C.. (2010). Skeletal muscle specific genes networks in cattle. Funct. Integr. Genomics 10, 609–618. 10.1007/s10142-010-0175-2 20524025 PMC2990504

[B32] Mudadu M. A. Porto-Neto L. R. Mokry F. B. Tizioto P. C. Oliveira P. S. N. Tullio R. R. (2016). Genomic structure and marker-derived gene networks for growth and meat quality traits of Brazilian Nelore beef cattle. BMC Genomics 17:235 10.1186/s12864-016-2535-3 26979536 PMC4791965

[B33] Pace J. M. Corrado M. Missero C. Byers P. H. (2003). Identification, characterization and expression analysis of a new fibrillar collagen gene, COL27A1. Matrix Biol. 22, 3–14. 10.1016/S0945-053X(03)00007-6 12714037

[B34] Page B. T. Casas E. Heaton M. P. Cullen N. G. Hyndman D. L. Morris C. A. (2002). Evaluation of single nucleotide polymorphisms in CAPN1 for associations with meat tenderness in cattle. J. Anim. Sci. 80, 3077–3085. 10.2527/2002.80123077x 12542147

[B35] Picard B. Lebret B. Cassar-Malek I. Liaubet L. Berri C. Le Bihan-Duval E.. (2015). Recent advances in omic technologies for meat quality management. Meat Sci. 109, 18–26. 10.1016/j.meatsci.2015.05.003 26002117

[B36] Ramayo-Caldas Y. Renand G. Ballester M. Saintilan R. Rocha D. (2016). Multi-breed and multi-trait co-association analysis of meat tenderness and other meat quality traits in three French beef cattle breeds. Genet. Sel. Evol. 48:37. 10.1186/s12711-016-0216-y 27107817 PMC4842279

[B37] Reverter A. Fortes M. R. S. (2013). Breeding and Genetics Symposium: building single nucleotide polymorphism-derived gene regulatory networks: towards functional genomewide association studies. J. Anim. Sci. 91, 530–536. 10.2527/jas.2012-5780 23097399

[B38] Saito R. Smoot M. E. Ono K. Ruscheinski J. (2012). A travel guide to Cytoscape plugins. Nat. Methods 9, 1069–1076. 10.1038/nmeth.2212 23132118 PMC3649846

[B39] Schroeder T. Tonsor G. James M. (2013). Beef Demand Determinant Study. Beef Checkoff. Available online at: http://www.beefboard.org/evaluation/130612demanddeterminantstudy.asp

[B40] Segura V. Vilhjálmsson B. J. Platt A. Korte A. Seren Ü. Long Q.. (2012). An efficient multi-locus mixed-model approach for genome-wide association studies in structured populations. Nat. Genet. 44, 825–830. 10.1038/ng.2314 22706313 PMC3386481

[B41] Shannon P. Markiel A. Ozier O. Balinga N. Wang J. Ramage D.. (2003). Cytoscape: a software environment for integrated models of biomolecular interaction networks. Genome Res. 13, 2498–2504. 10.1101/gr.1239303 14597658 PMC403769

[B42] Smith T. P. Casas E. Rexroad C. E. Kappes S. M. Keele J. W. Smith T. P. L.. (2000). Bovine CAPN1 maps to a region of BTA29 containing a quantitative trait locus for meat tenderness. J. Anim. Sci. 78, 2589–2594. 10.2527/2000.78102589x 11048924

[B43] Stanton H. Melrose J. Little C. B. Fosang A. J. (2011). Proteoglycan degradation by the ADAMTS family of proteinases. Biochim. Biophys. Acta Mol. Basis Dis. 1812, 1616–1629. 10.1016/j.bbadis.2011.08.009 21914474

[B44] Tizioto P. C. Decker J. E. Taylor J. F. Schnabel R. D. Mudadu M. A. Silva F. L.. (2013). Genome scan for meat quality traits in Nelore beef cattle. Physiol. Genomics 45, 1012–1020. 10.1152/physiolgenomics.00066.2013 24022219

[B45] Van de Werken R. Gennari M. Tavella S. Bet P. Molina F. Lin S.. (1993). Modulation of tensin and vimentin expression in chick embryo developing cartilage and cultured differentiating chondrocytes. Eur. J. Biochem. 217, 781–790. 10.1111/j.1432-1033.1993.tb18306.x 8223621

[B46] Xia J. Qi X. Wu Y. Zhu B. Xu L. Zhang L.. (2016). Genome-wide association study identifies loci and candidate genes for meat quality traits in Simmental beef cattle. Mamm. Genome 27, 246–255. 10.1007/s00335-016-9635-x 27126640

[B47] Yang C. K. Kim J. H. Stallcup M. R. (2006). Role of the n-terminal activation domain of the coiled-coil coactivator in mediating transcriptional activation by β-catenin. Mol. Endocrinol. 20, 3251–3262. 10.1210/me.2006-0200 16931570 PMC1770943

